# Editorial: Predictive modeling of cognition and behavior on quantum principles

**DOI:** 10.3389/fpsyg.2022.1107745

**Published:** 2023-01-09

**Authors:** Ilya A. Surov, Emmanuel E. Haven, Peter beim Graben, Sandro Sozzo, Catarina Moreira

**Affiliations:** ^1^ITMO University, Saint Petersburg, Russia; ^2^Memorial University of Newfoundland, St. John's, NL, Canada; ^3^Bernstein Center for Computational Neuroscience Berlin, Berlin, Germany; ^4^Università degli Studi di Udine, Udine, Italy; ^5^Queensland University of Technology, Brisbane, QLD, Australia

**Keywords:** quantum, ontology, meaning, perception–action loop, cognitive change

One of the biggest challenges for quantitative modeling in cognitive science and humanities is to find regularities in the human mind and in human behavior in general. Mechanistic-like theories of classical behaviorism and psychophysics apparently do not meet these challenges, leaving spontaneous and creative aspects of our nature out of scope. Conceptually different alternative, suitable to capture non-deterministic, subjective, and irrational regularities of human activity, is suggested by the formal framework of quantum theory when it is applied to cognition and decision-making, leading to the emerging field of quantum cognition.

By taking advantage of quantum-like structures of cognitive state spaces expressed in simple and coherent mathematical form, the quantum cognition approach succeeds in many modeling tasks that are typically highly problematic for traditional methods. Quantum cognition allows to build the quantitative models of irrational decisions and cognitive fallacies; the semantics of natural language; unexpected game equilibria and economic behavior; bioinformatics and artificial intelligence; and other areas.

However, many of the existing quantum-inspired models do not have wide practical applications due to their lack of predictive power. They provide conceptual explanations of behavioral and cognitive phenomena and succeed in the post-factum fitting of experimental results, but fail to prognose these results in advance. Achieving a level of predictive quality could drastically increase the practicality of quantum models in decision support systems whether they occur in settings such as economics, robotics, information retrieval, or other areas of modern technology.

The important pieces for resolving this puzzle are expected to be sourced from the modeling of cognitive mechanisms of prognosis, pro-active thinking, and decision- and meaning-making activities of living systems. Contributions to this Research Topic build a lot of those valuable connections, as shown in Figure 1. Interesting many-sided conversations arise in the ontology vs. epistemology perspective, both fundamental and applied, which is the most common theme across the papers.

**Figure 1:**
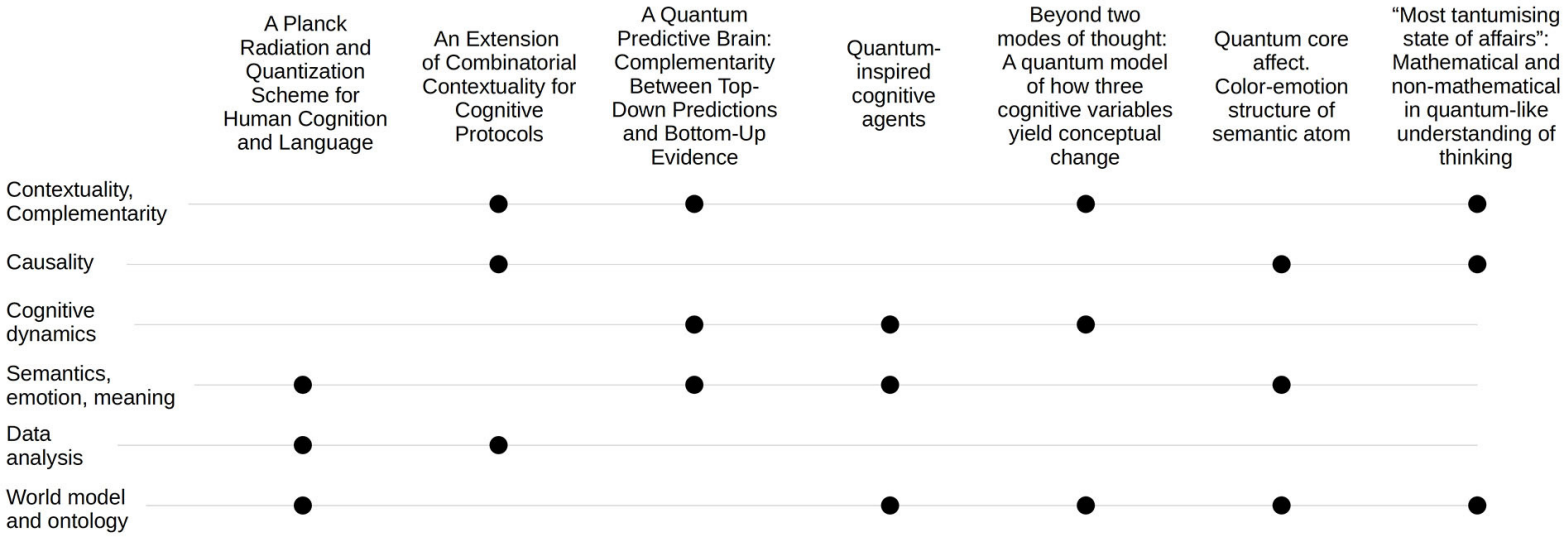
Distribution of the papers among major topics.

As a starting point in our review, Aerts and Beltran state the question on how statistics of word usage in natural language is constrained by the regularities of linguistic meanings. By reconstructing more than a century-old physical thought that later developed into quantum theory, the authors inquire whether similar procedures could work for human language. Among the three physically grounded theoretical laws for frequency-rank distributions, the words precisely follow the theory developed for the quanta of light. This distribution then provides a previously lacking foundation for the famous Zipf power law. The sharpness of this choice specifies a particular kind of *distinguishability* for conceptual meanings in human cognition, supporting the Brussels interpretation of quantum particles as *conceptual* entities. According to this view, the “substance of meaning” takes a central place within the ontology of nature as a fundamental feature of an individual's creative participation in the world.

This realistic position, however, is challenged by Plotnitsky. In his view, reality emerges from quantum measurement phenomena, but these phenomena themselves are not comprehensible and not realistic. He takes this century-old “reality without realism” view, ascending from Bohr to the cognitive domain. Cognitive information, obeying the laws of QT in parallel with the matter, then analogously loses ontological status giving rise to the “ideality without idealism”. This exotic stance, however, bears important insights into the consciousness–unconsciousness interplay and a qualitative side of nature beyond any representation.

Considered a practical model of the world, ontology is also at the heart of applied cognitive modeling. This aspect of ontology is considered most extensively in the study proposed by Huber-Liebl et al. Based on a perception–action cycle, the authors develop an elaborate cognitive architecture including dedicated functional modules responsible for subjective intentions, permissions, planning, communication, and interaction with the environment. The authors use this cycle to articulate the agent's cognitive simulation, interaction with the world, and communication with others. Formalized in the mathematical apparatus of QT, their model is shown to work in real navigation tasks, where a cognitive agent builds its own ontology of the experimental environment.

The perception–action cycle is essentially a cybernetic feedback loop, describing the interaction of living organisms with their environment in close analogy to von Uexküll's biosemiotics, where such a functional cycle generates a subjectively meaningful worldview—an *Umwelt*—being a predecessor of applied ontology in modern cognitive science. This cyclic structure is also central in Surov's model of subjective meaning. The author identifies the meaning of any information with a quantum qubit state, constructed by an individual in every act of a genuine decision. Visualized in spherical geometry, the qubit state space functions as a subjective world of a decision-maker—a task-specific Umwelt used to control this process. Remarkably, the ontology of this world appears to be that of emotion and color, as shown by comparison with classical results in the corresponding branches of cognitive science. Considered an atom of affective-semantic medium, it provides an alternative view on cognitive quantization proposed by Aerts and Beltran.

As a major element of the subjective worldview, the paper proposed by Winslow and Gabora focuses on the child's concept of the Earth as it evolves from flat to round to spherical. The authors establish that different steps of this development involve various modes of thought characterized by their abstractness, divergence, and context-specificity. Building on the quantum theory of concepts, these variables find formalization as dimensionality of quantum-theoretic cognitive space, properties of superposition states, and observables. Discontinuous reorganization of the concept of the Earth and an individual's place on it is enabled by the interplay of these three cognitive variables, extending the classical fast-slow and convergent-divergent modes of thinking.

The study proposed by Mastrogiorgio considers an analogous cognitive updating process, generalized to the arbitrary interaction of an agent with the world. The basis for such evolution is the classical principle of minimizing the difference between bottom-up evidence and top-down expectations in individual cognition, like the perception-action cycle used by Huber-Liebl et al. and Surov. The crucial novelty here is seeing this difference as a mismatch of an individual's and environment's cognitive-behavioral basis. This misalignment then finds natural formalization as an incompatibility of the corresponding quantum-theoretic observables. The conceptual shift achieved by changing the prediction basis is resonating with the case considered by Winslow and Gabora. Generally, as the author shows, this approach leads to a quantum-probabilistic extension of the energy minimization principle of natural thinking, yielding an advantage that well-known in the quantum cognition community.

Cognitive modeling pursued by Huber-Liebl et al., Mastrogiorgio, and Winslow and Gabora crucially depends on revealing situation-specific regularities in the agent's environment. Instruments for that are reported by Obeid et al. The authors describe methods for discriminating causal influences from genuine contextuality and noise in cognitive-behavioral data which is relevant for their correct representation and use. These methods are directly applicable, for example, to the statistics of word co-occurrence in natural language, suggesting an interesting intersection with the study proposed by Aerts and Beltran.

Summing up, in this Research Topic, we observe how quantum-inspired research enters new areas of linguistics, cybernetics, emotion modeling, cognitive development, and behavioral data analysis. In this foray, a few papers already approach the practical advantage that we attempted to foresee in our call for papers, while others prepare inspiring foundations for the future. We congratulate all contributors for making this progress and look forward to further development of their ideas.

## Author contributions

IS, EH, and PG prepared the text. All authors contributed to the article and approved the submitted version.

